# Feasibility, Acceptability, and Programme Effectiveness of Misoprostol for Prevention of Postpartum Haemorrhage in Rural Bangladesh: A Quasiexperimental Study

**DOI:** 10.1155/2014/580949

**Published:** 2014-04-07

**Authors:** Abdul Quaiyum, Rukhsana Gazi, Shahed Hossain, Andrea Wirtz, Nirod Chandra Saha

**Affiliations:** ^1^Centre for Reproductive Health, icddr,b, Bangladesh; ^2^Centre for Equity and Health Systems, icddr,b, Mohakhali C/A, Dhaka 1212, Bangladesh; ^3^Department of Epidemiology, The Centre for Public Health and Human Rights, Johns Hopkins Bloomberg School of Public Health, 615 North Wolfe Street/E7144, Baltimore, MD 21205, USA; ^4^Health Systems and Infectious Diseases Division, icddr,b, Bangladesh

## Abstract

We explored the feasibility of distributing misoprostol tablets using two strategies in prevention of postpartum haemorrhage (PPH) among women residing in the Abhoynagar subdistrict of Bangladesh. We conducted a quasiexperimental study with a posttest design and nonequivalent comparison and intervention groups. Paramedics distributed three misoprostol tablets, one delivery mat (Quaiyum's delivery mat), a packet of five standardized sanitary pads, and one lidded plastic container with detailed counseling on their use. All materials except misoprostol were also provided with counseling sessions to the control group participants. Postpartum blood loss was measured by paramedics using standardized method. This study has demonstrated community acceptability to misoprostol tablets for the prevention of PPH that reduced overall volume of blood loss after childbirth. Likewise, the delivery mat and pad were found to be useful to mothers as tools for assessing the amount of blood loss after delivery and informing care-seeking decisions. Further studies should be undertaken to explore whether government outreach health workers can be trained to effectively distribute misoprostol tablets among rural women of Bangladesh. Such a study should explore and identify the programmatic requirements to integrate this within the existing reproductive health program of the Government of Bangladesh.

## 1. Introduction 

Globally, postpartum haemorrhage (PPH) has been identified as one of the leading causes of maternal mortality and morbidity and approximately one-third of total maternal deaths occur in Asia [1]. We learned from both national DHS surveys [2, 3] and individual studies [4, 5] that haemorrhage has been one of the major causes of maternal deaths in Bangladesh during this last decade. PPH is unpredictable, catastrophic, and may occur even among women who are considered to be at low risk [6]. As a result, experts have concluded that the millennium development goals will not successfully be achieved without reducing deaths attributable to PPH, particularly those that occur in resource poor settings [7].

Several causes are attributable to the development of PPH, most commonly reported is uterine atony, as well as surgical incisions or lacerations and coagulation disorders [8]. Investigators of a study conducted in Pakistan identified two major causes of primary PPH: uterine atony (70.5%) and traumatic lesions of genital tract (29.4%) [9]. The investigators of this study further suggested that uterine atony was associated with augmented labor, prolonged labor, retention of placenta, and multiple pregnancies [9]. The consequences of primary PPH are serious in nature and may include hypovolemic shock, cerebral anoxia, renal failure, anemia, puerperal sepsis, and Sheehan's syndrome [10].

Although effective clinical treatments for PPH are available, these are not practical for use in developing countries where the majority of births occur at home and are overseen by the untrained attendants. Following literature reviews, we have learned that active management of third stage of labour, especially the administration of uterotonic drugs, may reduce the risk of PPH due to uterine atony without increasing the incidence of retained placenta or other serious complications [11]. Although active management of third stage of labour has proven efficacious in the management of PPH, the challenge is translating active management into general use in rural settings. For example, oxytocin and syntometrine are preferred uterotonic drugs but are not useful in the situation of home deliveries where parenteral administration is unsuitable and drug storage is a problem as it requires refrigeration [12].

An alternative drug, misoprostol, a prostaglandin, has been proven to be practical and effective in developing countries to reduce PPH [12–15]. Misoprostol is low cost, stable at room temperature, and easy to administer [16]. Misoprostol can be effectively administrated sublingually [17], orally [14, 18], or rectally [19, 20]. Researchers in prior studies have demonstrated some side effects of misoprostol, which include postpartum maternal shivering and fever while no side effects have been reported for the newborn [21]. The benefits of misoprostol, however, should be weighed against reported side effects, particularly in the resources poor settings where there is no other alternative.

In Bangladesh, wide geographical variation exists in the availability of emergency obstetric care facilities in the public sectors [22]. From a national survey done in both rural and urban areas of Bangladesh we learned that there are challenges related to health access and uptake and barriers related to economic cost; more than 75% of women experiencing delivery related complications such as convulsion or haemorrhage either failed to seek treatment or were treated by an unqualified provider [23]. Further exploration by researchers suggested that the principal reasons for not seeking care were related to concerns over medical costs and severe socioeconomic disparities [23]. Gross misunderstandings and misconceptions regarding PPH at the community level in Bangladesh also contribute to low access and uptake of care [24–26]. Among service providers, current methods to assess PPH are also suboptimal. In Bangladesh, visual assessments are used to estimate postpartum blood loss even in the clinical settings. By conducting a review of published studies, researchers have confirmed that visual assessments of postpartum blood loss significantly underestimate blood loss, compared to direct estimation and other methods [27]. Investigators in a randomized control trial done in India assessed that visual assessment was 33% less accurate than blood loss estimated by a blood collection drape [28]. In Bangladesh, the absence of any suitable tool available to identify a case of suspected PPH at the community level may result in delays to accessing health care facilities during obstetric emergencies.

Distribution of misoprostol for prevention of PPH has been identified as one of the most cost effective interventions for safe motherhood in resource poor settings [29]. A cost-effectiveness analysis has led experts to emphasize that training of traditional birth attendants (TBAs) to administer misoprostol for the treatment of PPH has the potential to both save money and improve the health of mothers in resource poor settings [30]. 

The objective of this study to test the feasibility, acceptability, and program effectiveness of misoprostol distributed by different cadres of health providers in the prevention of PPH after delivery of the baby. This was a collaborative effort by the International Centre for Diarrhoeal Disease Research, Bangladesh (icddr,b), the Obstetric and Gynaecological Society of Bangladesh (OGSB), and the government's Reproductive Health Program (RHP). In the same study researchers found that the use of a delivery mat (Quaiyum's mat) can guide women and community people about the amount of blood loss after delivery [31].

## 2. Materials and Methods

We conducted a quasiexperimental study with a posttest design and nonequivalent comparison and intervention groups. Research activities were conducted between July 2006 and December 2007 in Abhoynagar subdistrict of Jessore district of Bangladesh, which has a population of 240,000.

The study assessed two strategies for distribution of tablets (Table 1). In Strategy 1, trained study paramedics distributed misoprostol and provided counseling on use and potential side effects. In Strategy 2, distribution and counseling on misoprostol use were done by the participant's intended TBA. Three randomly selected subdistricts were assigned to two-distribution strategies or control area. Pregnancies in each union were identified and enumerated based on the expected date of delivery (EDD) falling between July 16, 2006 and May 2007. Other eligibility criteria included local residents, married women of reproductive age group, and no prior reported complications like heart diseases. Women with expected delivery dates between these dates and who met the other inclusion criteria were informed of the study activities and asked for their consent to participate. Those who gave an informed consent to participate were enrolled and followed for to 24 hours postdelivery. All participating women in the intervention areas (both distribution strategies) were provided with 600 *μ*g (3 tablets of 200 *μ*g) misoprostol oral doses. This dosage was recommended and found to be effective in previous research [29, 32, 33]. All enrolled women, their intended delivery attendant, and at least one nearest kin of the pregnant women were counseled on misoprostol use: how and when to take tablets, potential side effects, methods to remedy side effects, and danger of swallowing the tablets prior to delivery. Women were also counseled on the danger signs of pregnancy and delivery and were informed of where to go if danger signs appear. Counseling occurred at least three times during the course of follow-up: at enrollment, during follow-up visits, and 4 weeks before the expected date of delivery.

In Table 1, research activities are described for the three study areas. During the visit, 4 weeks before the expected delivery date, paramedics distributed three misoprostol tablets, one delivery mat (Quaiyum's delivery mat), a packet of five standardized sanitary pads, and one lidded plastic container with detailed counseling on their use. All materials except misoprostol were also provided in the control group participants. All women were provided with a study identification card that included the cell phone numbers of the study medical officer and field supervisors. The family member or attendant was reminded to save the used mat and pads after delivery in the lidded container until a study personnel came for collection. During the postdelivery visit (24 hours after delivery), the study paramedics weighed the used mat and pads by a calibrated digital scale. The study paramedics also conducted visual assessment of blood loss and completed the structured questionnaire to collect information on the delivery and misoprostol use. All complicated medical cases were referred to appropriate facilities.

The standard dry weight of a Quaiyum's delivery mat is 40 ± 2 grams and sanitary pads weigh 16 ± 1 gram. A fully soaked mat can retain 448.0 ± 58.2 mL of blood and a fully soaked pad may retain 60.2 ± 2.1 mL of blood. To estimate blood loss and identify possible cases of PPH, the soaked mat and all soaked pads were weighed after 24 hours of delivery using a calibrated, electronic postal scale prior to disposal. PPH was defined as blood loss >500 mL after birth, as per international protocol [32]. Blood loss was calculated by subtracting the dry weight of the mat and pads from total weight of the soaked mat and pads.

An additional qualitative assessment included subgroups of female participants (22), their husbands (22), and mothers-in-law (15) who were selected to take part in in-depth interviews. The female participants included women who experienced PPH (15) and women who did not experience PPH (7). Qualitative data was collected using an open-ended interview guide. In-depth interviews were audio recorded and later transcribed for content analysis. The study was approved by the Research Review Committee (RRC) and Ethical Review Committee (ERC) of icddr,b. All severe adverse effects were reported to the Data Safety Monitoring Board under the Ethical Review Committee of icddr,b. Two professors of obstetrics and gynaecology of Bangladesh served as technical advisers during study.

## 3. Results

### 3.1. Sociodemographic Characteristics of Participants

Sociodemographic characteristics of female participants are shown in Table 2. There were no major differences in characteristics of women in intervention A, intervention B, and control area. Overall, 17.1 to 22.4% women had no formal education. A higher proportion of husbands in control areas reported having no formal education compared to intervention areas A and B. The majority of the women in both intervention and the control areas were from low income families. Overall, two percent of the women in both areas were nulliparous.

### 3.2. History of Current Pregnancy and Delivery by Intervention and Control Areas

Table 3 displays pregnancy and delivery outcomes of participants during the study. In intervention areas, 22.7 to 38% women received ANC visits compared to 27.2% women in control area who received ANC. Few women experienced vaginal bleeding during the current pregnancy (0.6 to 1.8%). Significantly higher proportions of women in control area (49.5%) than intervention areas (41% in intervention A and 45.9% in intervention B) mentioned reported medication use to mitigate labor pains. Medication included intramuscular injections, intravenous infusion, and oral tablets. Irrespective of study area, most deliveries were assisted by traditional, untrained TBAs. In all study areas, approximately two percent of deliveries were still-births.

### 3.3. Feasibility of Distribution of Misoprostol Tablets and Its Acceptability to Women

**Figure 1:**
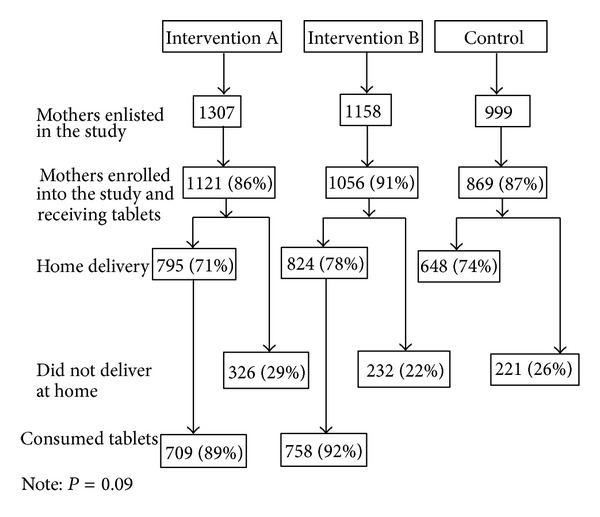
Distribution and consumption of misoprostol tablets for prevention of postpartum haemorrhage in intervention and control areas.

Figure 1 shows enrollment and distribution of misoprostol tablets in intervention and control areas. For instance, in intervention area A, 1307 pregnant women were identified, of which 1121 women were enrolled into the study, and 795 mothers delivered at home. A total of 709 (89%) mothers consumed misoprostol tablets in intervention area A. In intervention area B, 758 (92%) mothers took misoprostol tablets. There was no statistical significant difference between intervention area A and intervention area B (*P* = .09) regarding tablet consumption. Participants who did not take misoprostol tablets cited the following reasons: the placenta was out simultaneously with delivery of baby (13%), the person who had been informed about misoprostol tablets was absent at the time of delivery (33%), and somebody from the family was opposed to taking the tablets (41%).

### 3.4. Consumption of Misoprostol Tablets and Perceived Benefits

Irrespective of distribution of tablets by TBAs or paramedics (Strategy 1 or 2), there was no reported mistiming of tablet consumption by participants; all participants reported taking misoprostol tables postdelivery. Over 70% of the women consumed the misoprostol within 1 to 3 minutes after delivery. Another 15% women took it instantly after delivery. Among those who consumed the tablets, 80% believed that misoprostol tablets were beneficial to them. The benefits cited include placenta was expelled quickly (61.8%), bleeding was reduced (74.8%), pain was minimized (6.2%), and physical weakness was reduced (6.1%). Almost all (98.0%) of these participants indicated they would advise other pregnant women to use misoprostol tablets following delivery. The majority of the participants (85.8%) indicated willingness to buy tablets during future pregnancies.

During in-depth interviews with female participants, mothers-in-law, and husbands, participants discussed their perspectives on the use of misoprostol tablets. Almost all of the interviewed women including mother in-laws believed that misoprostol tablets were beneficial for a newly delivered mother. The commonly cited expressions were “as mother took the tablets,” “there was less bleeding,” “placenta came out quickly,” “mother recovered soon,” and “mothers felt well quickly after childbirth.” Most husbands viewed treatment by misoprostol as “one type of* prathomic *(primary) treatment at home.” Perceived benefits among husbands included that misoprostol was “free of cost,” and “saves life and money.”

### 3.5. Reported Side Effects of Misoprostol Tablets

Of 1459 intervention participants who took misoprostol tablets, 558 (38.2%) reported at least one side effect. Among those reporting any side effects, 75.4% women had shivering and 37.8% experienced low grade fever (Table 4). Few reported nausea (4.8%) and/or vomiting (8.6%). Of 10 participants who experienced shivering and needed any measures, seven women had this problem for 30 minutes or less and three women reported to have it for two hours or more. Among four participants who suffered from fever and needed any measures, two women had this for 30 minutes and another 2 women had this for one hour or more.

### 3.6. Measures Taken for Side Effects

Of total 558 women who reported having any of the side effects, only 13 required or sought any additional treatment or care; these included two participants consulted with doctors, one participant was self-referred to Thana Health Complex, one consulted neighbors, and another 9 women were self-referred to Khulna Hospital. Additional care resulted from shivering (76.9%, 10/13), fever (23.1%, 3/10), and combined shivering and fever (8%, 1/13). However, no women required hospitalization for any adverse effects. Among women, those who reported side effects but did not seek additional care (*n* = 545), mentioned that they didn't consider these as serious problems thus, didn't require any additional care.

### 3.7. Assessments on Amount of Blood Loss after Childbirth

Table 5 displays the postpartum blood loss across the three areas. Overall postpartum blood losses in intervention areas were significantly lower compared to control area. In Intervention area A, mean blood loss was 437.1 ± 171.2 (95% CI 425.1–448.9) and 478.1 ± 194.5 (CI 464.7–491.3) in intervention area B, compared to 486 ± 194.8 (CI 471.0–500.9) in control area (*P* < .001). The prevalence of PPH per area was 4.7% (38/794), 10.3% (85/824), and 12.5% (81/648) in areas A, B, and control, respectively.

### 3.8. Reported Benefits of Delivery Mat and Pad

Over 90% of the participants reported opined that delivery mat and pads had been beneficial during delivery.  Table 6 presents perceived benefits of the mat and/or pad. Frequently reported benefits included comfort (72.3%), no need of additional cloth (47.7%), and no need to wash the mat or pad (38.5%) (Table 6).

The participants of the qualitative component (women and their husbands) also provided perspectives on the benefits of the mat and pad. Most of the women indicated that the delivery mat and pads were “hygienic, clean, and soft.” Women preferred the delivery mat for the following reasons: the amount of blood loss was visible to them, they did not require extra clothes for collecting postpartum blood, and no washing was required. Husbands appreciated using delivery mat and pads because they thought “it kept the environment clean,” “it helped the village doctor to give proper treatment to the mother,” “quick decisions were made for hospitalization,” and “supply was free of cost.”

## 4. Discussion

In the present study, we explored the feasibility of distributing misoprostol tablets to prevent PPH among women residing in rural settings of Bangladesh. We further explored alternative methods of distribution, comparing distribution and counseling through paramedics with distribution and counseling through TBAs. Study findings suggest that community-based distribution of misoprostol tablets through minimally trained TBAs and paramedics can be a feasible strategy for reducing PPH in the resource poor settings and settings where there are limited health care facilities or absence of other alternatives.

Irrespective of the distribution strategy, the overwhelming majority (90%) of participants consumed the misoprostol, reflecting acceptability of the treatment. Based on the fact that misoprostol was consumed at the time directed by TBAs or paramedics, immediately following delivery (meaning no mistiming of consumption of tablets), we believe this study demonstrates the viability of employing TBAs and paramedics for such distribution efforts. Similar results have been demonstrated in settings comparable to Bangladesh; for example, investigators from a study in India reported that paramedical workers from rural primary health centers were able to administer oral misoprostol for active management of third stage of labor for prevention of PPH [18]. Another randomized double-blind placebo-controlled trial conducted in India has also confirmed that oral misoprostol can safely and effectively be distributed by skilled TBAs for home deliveries [33]. Community-based distribution such as this may also be cost effective as misoprostol is a lower-cost alternative to other PPH treatments, community-level distribution requires minimal training, distribution can be implemented outside of the clinical infrastructure, and community-level activities are already core components of TBA and paramedic responsibilities. Further research that would examine cost-effectiveness of misoprostol through alternative delivery methods might be beneficial.

This study adds to the body of knowledge on the benefits of the use of misoprostol to reduce PPH. When compared to either intervention area, participants of the control area experienced greater blood loss following childbirth. Derman and colleagues (2006) also found that misoprostol was associated with a decrease in mean postpartum blood loss and reported that one case of PPH was prevented for every 18 women treated [33]. Among female participants who consumed the misoprostol tablets, reported side effects were minimal but did include shivering and fever for which some sought additional care. Shivering and fever are known side effects of misoprostol and have been reported by other researchers [14, 34, 35]. In general, those who experienced the side effects considered these to be minor and the majority did not seek additional care. In fact, only 13 women sought medical attention for shivering and fever but no hospitalization was indicated for any of these side effects. Overall, among participants of the intervention areas and including those who developed PPH, about 80% of the participants perceived the treatment as beneficial for them. Qualitative findings supported this evidence: many women in intervention areas suggested that with consumption of the tablets, the placenta expelled quickly and bleeding was less. The majority of the women expressed willingness to misoprostol during future pregnancies and they would recommend it to others for use, reflecting positive perceptions and acceptability on the use and benefits of misoprostol tablets. Husbands and mothers-in-law, who are key individuals in decisions related to reproductive health, also supported these perceptions. Provision of misoprostol to all women who participated in the intervention areas of the study was not superfluous as it reduced overall volume of blood loss in these areas and may also confer secondary benefits. For example, reduction of volume of blood loss is also particularly important in the context of Bangladesh, where 49 to 50% of pregnant women were found to be anemic in rural areas [36, 37] and iron and folate status is found to be particularly low among women during the periconception period [38].

We also assessed the acceptability of misoprostol tablets and delivery mat and pads among women and their husbands. This study is a pioneer one that estimated blood loss after childbirth among women at the rural communities of Bangladesh using an easy blood collection method. Participants of the study felt that delivery mat and pads were very useful and convenient to them. With the ability to visually assess the amount of blood loss, collected by the delivery mat and pads, participants and family members believed they could make timely decisions of whether to seek care at formal health facilities. This increased capacity is extremely important in the context of rural Bangladesh where more than 80% of deliveries take place at home situations [3]. In a randomized control trial, the use of a medical drape to assess blood loss after delivery [18, 39] was demonstrably more efficient for measuring blood loss and identifying PPH, as compared to visual assessments [28]. However, the method requires trained personal for implementation and blood volume measurement. In Africa, one study reported that “Kanga”, rectangular cotton made fabric that is normally used as a skirt or head wrap or to carry a baby on a mother's back, was effectively used as a postpartum blood collection towel [29]. However, there must be standardized means to assess blood loss at the community level. In rural Bangladesh, a delivery mat will be very suitable because local women use similar materials, such as folds of old cloths, for the purpose of postpartum blood collection. Women found the delivery mat and pads useful and convenient for such use, as did the family decision makers (mother-in-law and husbands), who are important as these individuals must be motivated to support women's use of the delivery mat and pad.

One of the limitations of the study was our inability to track those female participants who delivered outside the study areas. There is a common tradition in Bangladesh that women usually deliver at their parental home, particularly with respect to the first child. Many women in our study were pregnant for the first time, so we were unable to follow those women through delivery. A second limitation was the use of a nonequivalent control group; this was due to scarcity of resources, though we do not believe nonequivalency changes the findings of the feasibility of misoprostol distribution.

## 5. Conclusions

This study highlighted the programmatic feasibility of distributing misoprostol tablets for prevention of PPH, through paramedics as well as TBAs in rural communities of Bangladesh. This study has demonstrated acceptability to misoprostol tablets for the prevention of PPH, and participants perceived these as generally beneficial to their health. Likewise, the delivery mat and pad were found to be useful to mothers as tools for assessing the amount of blood loss after delivery and informing care-seeking decisions. For scale-up of such programs, we recommend that TBAs should be properly educated on the correct administration and common side effects of this drug. Future research should explore the possibility of misoprostol distribution via TBA delivery kits during future PPH intervention studies. As we found, some family members were not convinced of the benefits of misoprostol tablets and prevented women from taking the tablets; it is suggestive that behavioral change and communication (BCC) activities should complement misoprostol distribution to build awareness among community members, particularly among husbands and in-laws. Further studies should be undertaken to explore whether government outreach health workers can be trained to effectively distribute misoprostol tablets among rural women of Bangladesh. Such a study should explore and identify the programmatic requirements to integrate this within the existing reproductive health program of the Government of Bangladesh.

## Figures and Tables

**Table 1:** Project activities by study areas.

Activity	Strategy 1	Strategy 2	Control
Distribution of mat and pad with counseling on methods of use and preservation	Conducted by project staff (paramedics). Provided to women, family members, and intended birth attendant	Conducted by the intended TBAs. Provided to women and family members	Conducted by project staff (paramedics). Provided to women, family members, and intended birth attendant

Distribution of misoprostol tablets and detailed counseling on misoprostol use, side effects, and remedies	Conducted by paramedics	Conducted by intended TBAs	No misoprostol tablets distributed

Measurements of blood loss within 24 hours of delivery	Conducted by paramedics		

Interview of participants	Conducted by project staff members		

Establishment of referral mechanism for complications	Standard for all sites		

**Table 2:** Profile of the mothers by areas.

	Intervention area-A *n* = 795 %	Intervention area-B *n* = 824 %	Control area *n* = 648 %
Age in years			
<20	22.1	26.5	20.5
20–24	33.5	33.1	32.4
25–34	40.0	35.7	40.7
35+	4.4	4.7	6.3
Women's education			
No education	22.4	17.1	20.1
1–5	30.9	32.2	33.5
6–10	44.4	47.7	45.7
11+	2.3	3.0	0.8
Husbands education			
No education	26.7	28.4	35.6
1–5	30.8	33.1	29.5
6–10	35.8	31.6	29.5
11+	6.7	6.9	5.4
Monthly family income in Taka (1 USD = 51 Taka)			
<4000 Taka	74.0∗	74.5∗	82.2
4000 and above	23.6∗	24.9∗	8.1
Do not know	2.4	0.6	9.7
Parity of the mothers			
0	1.8	1.7	1.8
1	48.4	47.9	45.0
2	26.2	27.8	31.1
3	13.2	14.9	11.4
4	7.0	4.0	6.6
5+	3.4	3.7	4.2

Note: ∗shows statistical significant difference between intervention and control; *P* < 0.001 at 95% level.

**Table 3:** Current pregnancy and delivery related characteristics.

	Intervention area-A *n* = 795 %	Intervention area-B *n* = 824 %	Control area *n* = 648 %
Duration of pregnancy in weeks			
35 to 39	39.7	35.0	40.9
40	40.8	43.4∗	33.8
41 to 44	19.5	21.6	25.1
Women received ANC	38.0∗	22.7	27.2
Women experienced vaginal bleeding during pregnancy	1.8	1.8	0.6
Labour pain was augmented by medication	41.0	45.9	49.5∗
Types of birth attendants			
Relatives	3.9∗	1.2∗	10.0
Untrained birth attendant	54.6	50.6	49.5
Trained birth attendant	38.6	47.2∗	38.0
Nurse/midwife	1.9	0.7	0.8
No birth attendant	1.0	0.2∗	1.7
Outcome of pregnancy			
Live birth	97.9	98.2	97.7
Still birth	2.1	1.8	2.3

Note: ∗shows statistically significant difference between intervention and control; *P* < 0.001 at 95% level.

**Table 4:** Side effect experienced by mothers who took misoprostol.

	Intervention area-A *n* = 281	Intervention area-B *n* = 277	Total *n* = 558
Nausea	5.3	4.3	4.8
Shivering	77.9	72.9	75.4
Vomiting	8.9	8.3	8.6
Low grade fever	50.5	24.9	37.8

Note: multiple responses considered.

**Table 5:** Assessments on amount of blood loss after childbirth.

	Intervention area-A (mean ± SD)	Intervention area-B (mean ± SD)	Control area (mean ± SD)
Mean blood loss in three areas	437.1 ± 171.2*	478.1 ± 194.5*	486 ± 194.8
95% CI	425.1–448.9	464.7–491.3	471.0–500.9

Mean blood loss among mothers who developed PPH in three areas	678.4 ± 213.7	684.7 ± 193.9	689.8 ± 186.1
95% CI	628.6–727.3	649.3–720.6	654.5–725.5

Note: ∗shows statistical significant difference between intervention and control; *P* < 0.001 at 95% level.

**Table 6:** Perceived benefits of delivery mat and pads supplied among women.

	Intervention area-A *n* = 739	Intervention area-B *n* = 795	Control area *n* = 625	Total *n* = 2159
No need to wash	33.0	43.6	38.9	38.6
Comfortable	79.5	65.5	82.2	75.2
No need for extra clothes	38.7	56.1	47.0	47.5
Easy to move/walk	21.8	18.4	16.6	19.0
Hygienic/safe	20.7	18.5	5.9	15.6
All blood remains in one place	8.4	2.0	3.5	4.6
Amount of blood is assessable	19.1	15.1	27.5	20.1
Keep body warm	1.2	0.9	2.7	1.5

Note: multiple responses considered.
